# Ketogenic Diet Improves Core Symptoms of Autism in BTBR Mice

**DOI:** 10.1371/journal.pone.0065021

**Published:** 2013-06-05

**Authors:** David N. Ruskin, Julia Svedova, Jessica L. Cote, Ursula Sandau, Jong M. Rho, Masahito Kawamura, Detlev Boison, Susan A. Masino

**Affiliations:** 1 Neuroscience Program, Trinity College, Hartford, Connecticut, United States of America; 2 Department of Psychology, Trinity College, Hartford, Connecticut, United States of America; 3 Robert Stone Dow Neurobiology Laboratories, Legacy Research Institute, Portland, Oregon, United States of America; 4 Alberta Children’s Hospital, Departments of Pediatrics and Clinical Neurosciences, University of Calgary Faculty of Medicine, Calgary, Alberta, Canada; 5 Department of Pharmacology, Jikei University School of Medicine, Tokyo, Japan; Université Pierre et Marie Curie, France

## Abstract

Autism spectrum disorders share three core symptoms: impaired sociability, repetitive behaviors and communication deficits. Incidence is rising, and current treatments are inadequate. Seizures are a common comorbidity, and since the 1920’s a high-fat, low-carbohydrate ketogenic diet has been used to treat epilepsy. Evidence suggests the ketogenic diet and analogous metabolic approaches may benefit diverse neurological disorders. Here we show that a ketogenic diet improves autistic behaviors in the BTBR mouse. Juvenile BTBR mice were fed standard or ketogenic diet for three weeks and tested for sociability, self-directed repetitive behavior, and communication. In separate experiments, spontaneous intrahippocampal EEGs and tests of seizure susceptibility (6 Hz corneal stimulation, flurothyl, SKF83822, pentylenetetrazole) were compared between BTBR and control (C57Bl/6) mice. Ketogenic diet-fed BTBR mice showed increased sociability in a three-chamber test, decreased self-directed repetitive behavior, and improved social communication of a food preference. Although seizures are a common comorbidity with autism, BTBR mice fed a standard diet exhibit neither spontaneous seizures nor abnormal EEG, and have increased seizure susceptibility in just one of four tests. Thus, behavioral improvements are dissociable from any antiseizure effect. Our results suggest that a ketogenic diet improves multiple autistic behaviors in the BTBR mouse model. Therefore, ketogenic diets or analogous metabolic strategies may offer novel opportunities to improve core behavioral symptoms of autism spectrum disorders.

## Introduction

Estimates indicate that autism spectrum disorder (ASD) affects at least 1 in 160 individuals [1]. Diagnosis involves three core symptoms: impaired social interactions, stereotyped repetitive behaviors and communication deficits [2]. Effective treatments are lacking for these core symptoms, and pharmacological approaches often target co-morbidities in the hope that relieving associated medical issues will yield more general improvements [3]. Seizures and/or EEG abnormalities are common [4], [5], and children with intractable seizures and autism suffer poor outcomes – even with aggressive interventions such as brain surgery [6]. Hence, it is of paramount importance that new, safe and effective treatment options be developed for ASD.

An effective historical metabolic treatment for refractory epilepsy is a high-fat, low-carbohydrate ketogenic diet (KD). This metabolic therapy experienced a resurgence over the past two decades, and multiple retrospective and prospective studies have confirmed its ability to dramatically reduce seizures in both children and adults [7], [8]. At a biochemical level, the KD forces a switch to predominant metabolism of ketones rather than glucose, and hallmark metabolic effects of the diet include increased blood ketones, reduced blood glucose, and increased mitochondrial function. Given the ubiquitous presence of metabolic abnormalities in neurological disorders [9], including autism [10], metabolism-based therapies such as the KD are of great interest - particularly in those diseases with a dearth of effective options. Furthermore, several putative mechanisms mobilized by the KD may alleviate autistic symptoms [11]–[13]. This rationale is supported by a thus-far singular prospective clinical report, a pilot study conducted on 30 children four to ten years old [14]. The diet was applied intermittently (four weeks on and two weeks off) for six months, and parents evaluated behavior using the Childhood Autism Rating Scale. Of those children who maintained the diet (18/30), 11% had significant improvement, 44% average improvement, and 44% mild improvement. In this initial study, the biggest improvements were noticed in those patients who showed only mild autistic behavior. Ketogenic diet therapy for epilepsy is applied continuously, and improvement potential of a standard (non-intermittent) regimen in autism has not been explored prospectively.

The BTBR T+ tf/j (BTBR) strain of mice, developed through the early and mid-20^th^ century, was recently characterized as having an autism-like behavioral phenotype [15]. These mice have low sociability in a number of tests, reduced communication, and elevated self-directed behavior compared to typical strains. Here we tested behavioral effects of a KD in this strain, and also tested for spontaneous seizures, abnormal EEG, and altered seizure threshold. We found that the KD improved all three behavioral deficits significantly [16], and, in contrast to other models of autism [17], [18] the BTBR mouse does not exhibit spontaneous behavioral or electrographic seizures. Furthermore, BTBR mice had minor and inconsistent changes in seizure threshold compared to control mice. Thus, behavioral improvement initiated by the KD was independent of its well-known antiseizure effects.

## Materials and Methods

### Ethics Statement

All procedures were performed in accordance with the NIH Guide for the Care and Use of Laboratory Animals, and approved by the Institutional Review Boards of Trinity College (A3869–01) and the Legacy Research Institute (A3234–01).

### Animals

At weaning, male BTBR T+ tf/j (Jackson Laboratories, Bar Harbor, ME) or C57Bl/6 littermates were housed socially (3–6 per cage). At five weeks of age, cages were assigned randomly to control diet (CD; LabDiet 5001, W.F. Fisher & Son, Somerville, NJ) or KD (F3666; BioServ, Frenchtown, NJ) fed ad libitum. All testing occurred at 8–10 weeks of age, i.e. between 3–5 weeks of dietary treatment. All measures were taken to minimize animal suffering.

### Behavioral Analysis

To test sociability, a Plexiglas apparatus measuring 22-x-42.5-x-19 cm with three equal chambers was used. Small wire cages were placed in the side chambers. Testing occurred as described previously [15]. Testing occurred in three 10 min phases with free movement among the three chambers. During phase one, the wire cages were vacant (a test for side bias). In phase two, a “stranger” mouse (C57Bl/6) was placed in the wire cage of one side chamber (a test of sociability). During phase three, the first (now familiar) C57Bl/6 mouse remained in place and a new “stranger” C57Bl/6 mouse was put in the other wire cage (a test for preference for social novelty). Stranger mouse placement was counterbalanced between tests. Time spent in each chamber and frontal contact (nose/face/forepaw contact with the cages and/or stranger mice) were quantified.

Self-directed repetitive behavior (self-grooming) was quantified in the three-chamber sociability test and during a separate 10 min test with mice alone in a small (19-x-29-x-12.5 cm) empty cage on a different day.

Communication was assessed by social transmission of a food preference as described previously [15]. Mice were habituated to eating KD or powdered CD, as appropriate, from glass jars (Dyets, Inc., Bethlehem, PA). A demonstrator mouse was fasted for 18 h, and presented with one jar of powdered flavored food (“trained” flavor) for 2 h. The demonstrator was returned to the home cage for 30 min to interact with cage-mate observer mice. Observer mice were fasted for 18 h, and then presented with both the “trained” flavor (eaten by the demonstrator) and an “untrained” flavor (novel flavored food) for 2 h. Jars were weighed before and after presentation. Flavor pairs were cocoa (2%) v. cinnamon (1%) and cinnamon (1%) v. cumin (0.25%).

### Blood Chemistry

Several days after completing all behavioral testing, a subset of mice was anesthetized with isoflurane for tail blood collection. Whole blood was analyzed for glucose and β-hydroxybutyrate with Precision Xtra meters (Abbott Laboratories, Bedford, MA).

### Intrahippocampal EEG and Seizure Threshold

EEG electrodes were implanted into BTBR and C57Bl/6 mice at 8 weeks of age. Briefly, bipolar stainless steel electrodes (insulated except 80–100 µm at the tip; tip diameter 5 µm; vertical tip separation 200–250 µm; Plastics One Inc., Roanoke, VA) were implanted bilaterally into hippocampal CA3 using stereotaxic coordinates (AP = −2.18 mm; ML = ±2.65 mm; DV = −2.6 mm to Bregma). A screw electrode was placed over frontal cortex and a ground electrode over the cerebellum. Electrodes were secured with dental cement. After a 3 d recovery period, animals were recorded continuously for 72 h.

Electrical brain activity was monitored using a Nervus EEG recording system and a Magnus 32/8 Amplifier (Nervus, Taugagreining, Iceland), and filtered (high-pass 0.3 Hz, low-pass 100 Hz). The digital EEG signal was analyzed using the LabChart version 7 software (AD Instruments, Colorado Springs, CO). An observer unaware of genotype assessed EEG records for seizure activity, defined by high-amplitude rhythmic discharges lasting for more than 5 s (repetitive spikes, spike-and-wave discharges, or slow waves).

Separate groups of mice were tested for seizure susceptibility (one test per mouse) in one of four seizure models. For electrical stimulation, after ocular local anesthesia (0.5% tetracaine drops), seizures were induced via corneal electrodes with a 3 s train of 2 ms duration pulses delivered at 6 Hz (Ugo Basile, Comerio, Italy). Mice were placed immediately in a standard cage and observed for 15–20 s for presence or absence of a seizure. Seizure threshold was determined with the “up/down” method [19]. For drug-induced seizures, we administered flurothyl (inhaled putative GABA antagonist; Sigma-Aldrich, St. Louis, MO), pentylenetetrazole (PTZ; GABA antagonist; Sigma-Aldrich, St. Louis, MO) or SKF83822 (dopamine D_1_ agonist; Tocris Bioscience, Bristol, UK). Flurothyl was infused (20 µl/min) into a Plexiglas chamber (15-x-20-x-35 cm) and latency to tonic/clonic seizure was recorded. PTZ was dissolved in saline and injected (i.p.; 50 mg/kg) and mice were observed for 30 min; peak seizure score was recorded according to the modified Racine scale. SKF83822 was dissolved in 5% dimethylsulfoxide in water and injected (s.c.; 2.0 mg/kg) and mice were observed for 80 min; latency to and number of seizures were recorded.

### Statistical Analysis

Social and grooming behavior were scored from video by two independent scorers, at least one of whom was blind to treatment. Inter-rater reliability (Cronbach’s α) for social behavior was 0.998, and for grooming was 0.997. Sociability data were non-normal and converted to ranks before analysis. Data were analyzed using t-test or ANOVA with Newman-Keuls post-hoc tests, except for 6 Hz testing which was analyzed according to Dixon and Mood [19]. Data are reported as mean ± standard error. *p*<0.05 was considered significant.

## Results

After 3–5 weeks on the KD, BTBR mice showed significant hallmark blood chemistry changes, i.e., having ketonemia (CD: 0.14±0.02 mM v. KD: 1.41±0.19 mM, p<0.001) and lowered glucose (CD: 142±5 mg/dL v. KD: 93±7 mg/dL, p<0.001).

**Figure 1:**
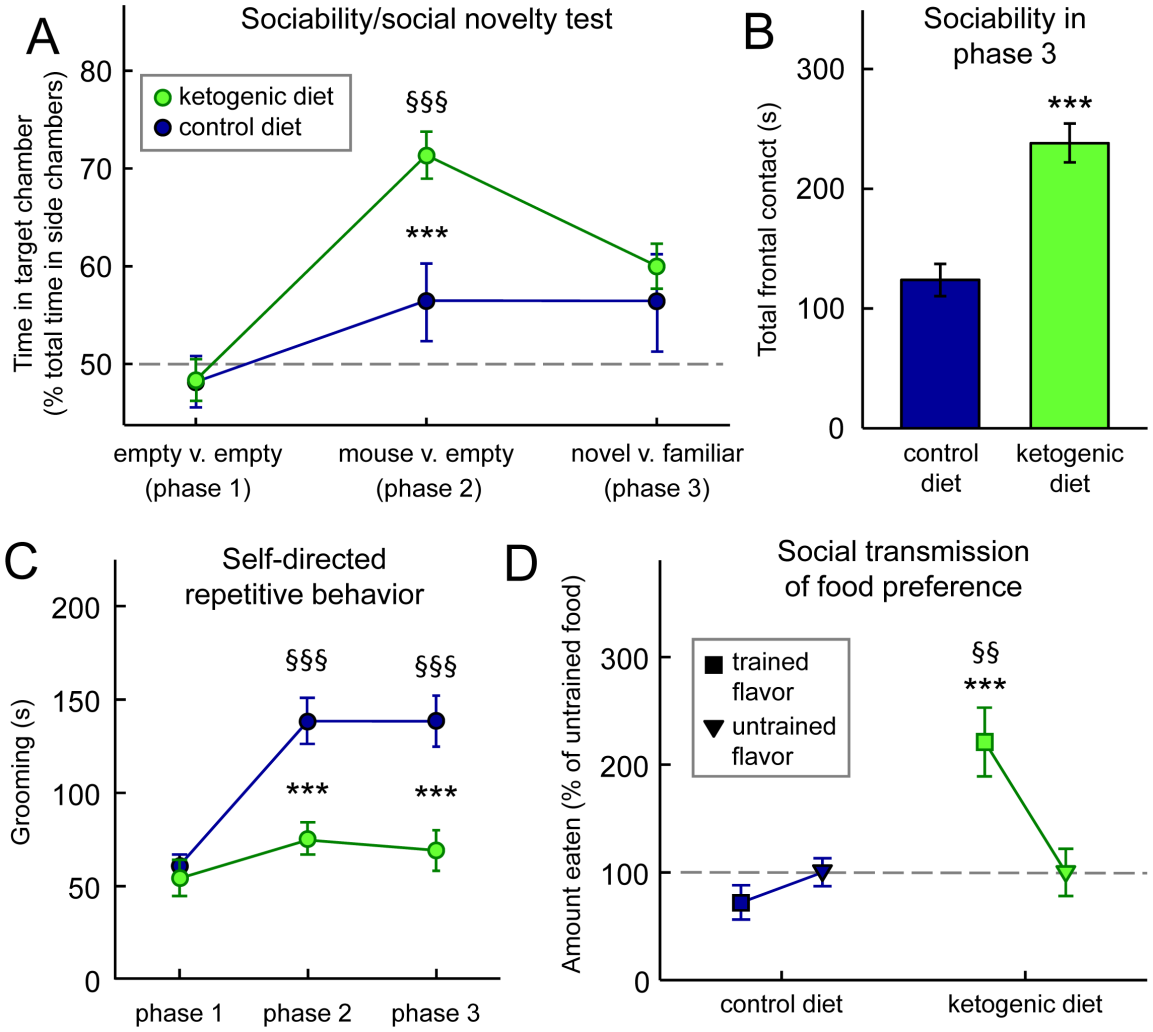
A KD reduces symptoms of autism in BTBR mice. (**A**) KD increases social interactions in the three-chamber test of sociability. During phase 2, CD-fed mice did not spend significantly more time with a mouse-containing versus an empty chamber (middle). However, KD-fed mice preferred spending time in the chamber with a mouse (middle). During phase 3, diet did not affect preference for social novelty (right). Diet F_(1,112)_ = 8.9, p<0.01; Social situation F_(2,112)_ = 12.5, p<0.001; Diet-by-Social situation interaction F_(2,112)_ = 3.2, p<0.05; n = 28–30. (**B**) KD feeding increases sociability as assessed in phase 3 of (**A**). Although there was no diet-related preference for social novelty, there was a significant diet-related difference in total time spent in frontal contact with the small wire cages, both of which contained mice. KD-fed mice spent significantly more time in frontal contact. n = 28–30. (**C**) KD feeding had no effect during phase 1, but resulted in significantly less self-directed repetitive behavior in phases 2 and 3 as assessed by time spent grooming. Diet F_(1,111)_ = 22.1, p<0.001; Phase F_(2,111)_ = 17.1, p<0.001; Diet-by-Phase interaction F_(2,111)_ = 6.8, p<0.01; n = 28–30. (**D**) KD feeding improves communication as assessed by the transmission of a food preference through social interaction; KD-fed mice ate significantly more of the trained flavor. Diet F_(1,33)_ = 42.5, p<0.001; Flavor F_(1,33)_ = 2.6, n.s.; Diet-by-Flavor interaction F_(1,33)_ = 6.8, p<0.05; n = 17–18. ***p<0.001, CD v. KD; ^§§^p<0.01, ^§§§^p<0.001 v. baseline (phase 1).

In the three-chamber test of sociability, BTBR mice fed either a CD or KD showed no inherent side preference (Fig. 1A, phase 1). As expected, BTBR mice were poorly social: when choosing between an empty chamber and a mouse-containing chamber, CD-fed BTBR mice showed no significant sociability (Fig. 1A, phase 2). However, KD-fed BTBR mice were significantly and robustly social when given this choice (Fig. 1A, phase 2). KD did not influence preference for social novelty (Fig. 1A, phase 3). The KD had no significant effect on sociability or preference for social novelty in highly social C57Bl/6 control mice (data not shown).

Upon further analysis of phase 3, we found that time spent in frontal contact with the mouse-containing cages in phase 3 was increased significantly in KD-fed mice (Fig. 1B). Therefore, treatment with a KD improved sociability in phases 2 and 3 of the three-chamber test as assessed by chamber time and frontal contact time, respectively.

In addition to increasing sociability in the three-chamber test, KD-feeding decreased self-directed repetitive behaviors as quantified by time spent grooming. In the absence of other mice (phase 1) there was no diet-related difference in grooming (Fig. 1C). After introducing other mice (phases 2 and 3), however, grooming time increased in CD-fed BTBRs, yet did not change in KD-fed animals. Thus the KD mice spent less time on this self-directed repetitive behavior than CD-fed during phases 2 and 3. In single small chamber tests of grooming, KD feeding did not significantly influence the time spent grooming (CD: 142±14 s; KD: 126±19 s, n.s.). Therefore, the KD appears to significantly reduce self-directed behavior in social but not in non-social situations in BTBR mice. The KD did not significantly affect grooming in control mice in the 3-chamber or single chamber tests (data not shown).

The KD enhanced social communication of food preference in BTBR mice. KD-fed BTBR mice ate significantly more of the “trained” detected previously on a littermate (demonstrator; Fig. 1D). CD-fed BTBR mice, as expected, showed no flavor preference. Therefore, in the BTBR mouse, the KD improved sociability and communication, and reduced self-directed repetitive behavior – thus alleviating significantly all three core hallmarks of ASD. The KD did not significantly affect social transmission of food preference in control mice in the 3-chamber or single chamber tests (data not shown).

**Figure 2:**
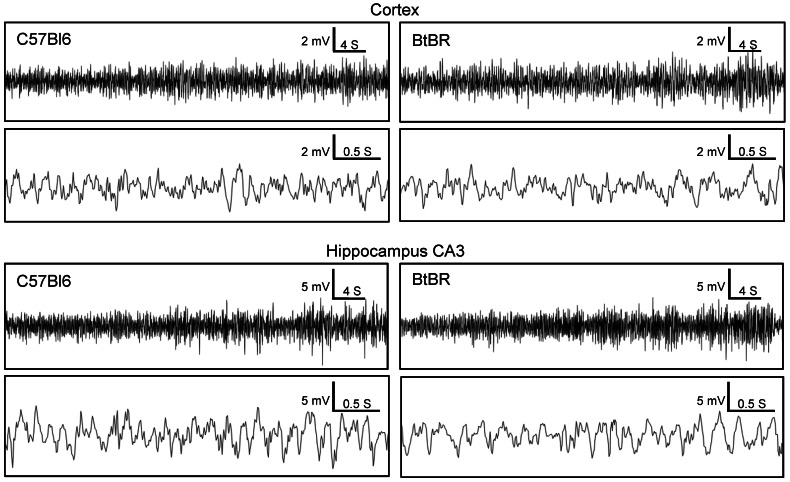
BTBR mice have normal electrical brain activity comparable to control mice. Cortical and intrahippocampal CA3 EEG traces from BTBR (right panels) and control C57Bl/6 (left panels) mice (n = 4/strain) recorded continuously for 72 h. Upper traces within each brain region are representative recordings of a 1 min period; lower traces are a higher resolution excerpt of the first 4 s from the respective upper trace. Throughout the 72 h recording period there was no evidence of electrographic seizures or any other pathological brain activity as determined by comparing the spike pattern, amplitude and frequency between the BTBR and C57Bl/6 EEGs.

Although seizures and/or abnormal EEG are often comorbid with autism, seizures have never been reported in BTBR mice, and we never observed an overt seizure during videotaped testing, routine handling, or within the home cage (data not shown). The absence of behavioral seizures, however, does not eliminate the possibility of electrographic abnormalities or altered seizure thresholds. For example, mouse models that show neither behavioral seizures nor abnormalities with scalp electrodes do show spontaneous electrographic seizures when assessed with intrahippocampal electrodes [20]. Here we did not find any evidence of electrographic seizures in hippocampal CA3 region in BTBR mice (or, as expected, in control C57Bl/6 mice; Fig. 2).

**Figure 3:**
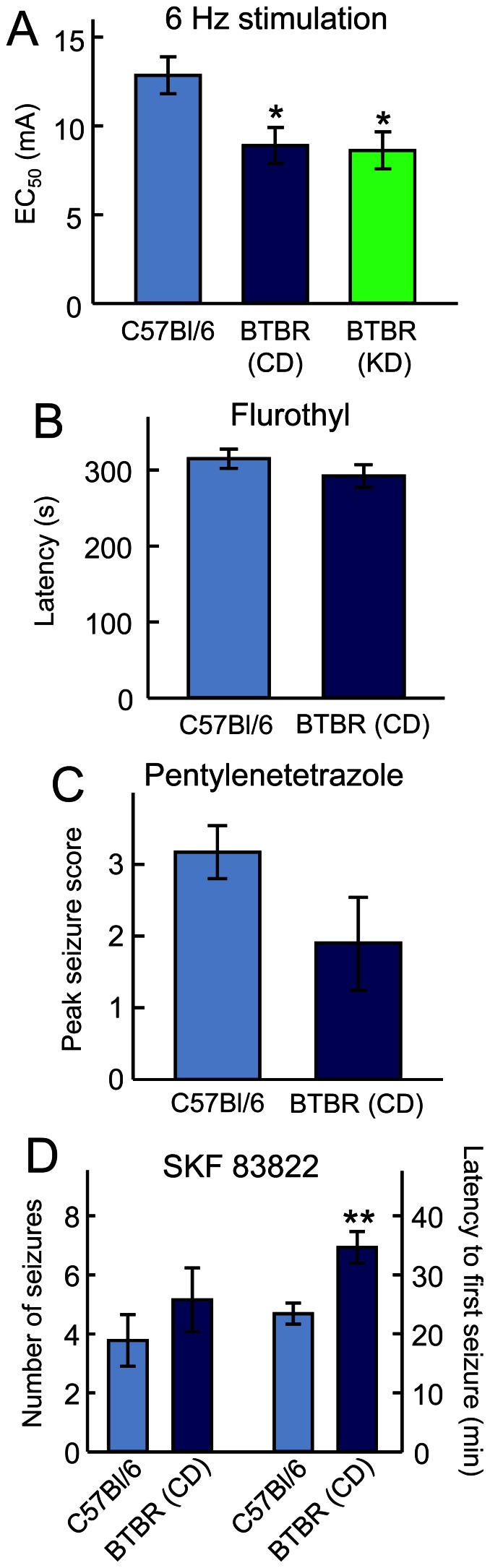
BTBR mice exhibit a varied seizure phenotype. (**A**) In response to 6 Hz electroconvulsive shocks, BTBR mice had a significantly reduced threshold compared to control mice, and this reduction was unaffected by KD feeding. *p<0.05 compared to control; n = 16 CD group, n = 8 KD group. (**B**) BTBR mice did not differ from control mice in latency to tonic/clonic seizures during flurothyl exposure; n = 7–8. (**C**) Peak seizure intensity was not different between BTBR and control mice in response to PTZ injection; n = 10–12. (**D**) Latency to first seizure after injection of the dopamine agonist SKF83822 was reduced in BTBRs, but the number of seizures did not differ. **p<0.01 compared to controls; n = 9–13.

To assess seizure susceptibility, we used acute electrical and chemical seizure tests in BTBR and control mice (Fig. 3). In the 6 Hz test, BTBR mice were more sensitive; however, three weeks of KD-feeding did not alter this threshold (Fig. 3A). BTBR mice were not different from control mice in their sensitivity (latency) to flurothyl (Fig. 3B) or their response to PTZ-induced seizures (Fig. 3C), demonstrating consistent results between these two GABA antagonists. Finally, BTBR mice were less sensitive (longer latency) to seizures induced by the dopamine D1 agonist SKF83822. BTBR mice thus have inconsistent changes in seizure threshold and a provocation-dependent seizure phenotype. Furthermore, in the one test with a decreased seizure threshold (6 Hz) the KD did not influence this susceptibility (Fig. 3A).

## Discussion

Here we show that the KD improves behavioral symptoms of ASD in BTBR mice – a model of autism that presents the three core deficits of ASD: reduced sociability and communication, and increased repetitive behavior. These results represent the first time that an established and effective dietary intervention demonstrates significant behavioral benefits in a mouse model of autism. Further, we demonstrate that BTBR mice do not have spontaneous behavioral or electrographic seizures and show minor and inconsistent changes in seizure threshold compared to control mice. Thus these findings characterize the seizure phenotype – i.e. lack thereof - of the BTBR mouse, and dissociate the behavioral changes in this mouse model of autism from the prevalent comorbidity of seizures or abnormal EEGs found in patients with autism. We further show that the KD does not alter seizure susceptibility to 6 Hz stimulation, the only test where seizure threshold was lower in BTBR mice. Thus, the beneficial behavioral effects of the KD are not secondary to its well-known efficacy against epilepsy and seizure activity. Furthermore, the KD did not influence sociability or repetitive behavior in control mice, thus ruling out non-specific effects on these behaviors associated with autism.

Despite this dissociation from the KD’s anti-seizure properties, mechanistic changes induced by the KD - some postulated to underlie its ability to reduce seizures - might be responsible for behavioral improvements. These mechanisms include the ability of the KD to reduce reactive oxygen species [21], [22], and inflammation [23]–[26], both noted to be increased in autism [27], [28]. Other inhibitory mechanisms that could reduce seizures and address anxiety – both comorbidities in autism - include increased neuronal inhibition, possibly via reduced glutamate release [29], [30], K_ATP_ channel activation [31]–[33], or increased GABAergic or adenosinergic inhibition [34], [35]. Recent investigations of the KD and autism have suggested that an increase in the inhibitory neuromodulator adenosine could yield a multiplicity of effects, including improved sleep, and reduced seizures, sensory disturbances and anxiety [20], [36], [37]. At this juncture, however, it is unknown how key metabolic changes produced by the KD – i.e., increased ketone production, reduced glucose, and increased energy capacity – might causally affect behaviors associated with ASD and more research is needed.

Beyond hypothesized antiseizure mechanisms of the KD, it is possible that some of the present results arise from a KD-related social anxiolytic effect. Mild stress increases grooming [38] and an increase in grooming by CD-fed BTBR mice in the presence of an unfamiliar mouse could signal social anxiety. BTBR mice are known to be more anxious than other strains in tests involving other animals, but not in more common tests for open space-related anxiety [39]. Studies of the KD in rats and other mouse strains have found varying results using these latter tests, but in humans, overall anxiety scores are improved with a KD [40], [41]. In general, amelioration of anxiety by the KD could play a role in normalizing social interactions in the three-chamber test. However, we provide some general evidence that reduced anxiety was not the only factor. First, the KD did not affect behavior in C57Bl/6 mice. Second, social transmission of food preference was also improved in BTBR mice by KD-feeding. Because this test involves communication among familiar cage-mates it involves presumably low-anxiety interactions. Regardless, anxiety is common in ASD [42] and an anxiolytic effect of the KD could be beneficial.

Diverse diet-based therapies are popular for ASD, but clear clinical evidence confirming efficacy is often lacking [43]. Here, ASD symptoms were alleviated in response to a KD, a treatment with clear – and sometimes dramatic - efficacy in epilepsy [7], [8], [44]. These results comport with published predictions [11], [12] and one report that an intermittent KD improved behavior in children with autism as quantified by the Childhood Autism Rating Scale [14]. Nevertheless, the present study is the first to demonstrate improved symptomatology due to a medically established dietary treatment in a mouse model of autism.

Based on these results, additional research on KDs or analogous metabolism-based strategies should be considered as we continue to seek novel and effective treatments for ASD. Immediate opportunities present themselves when ASD is co-morbid with a challenging case of epilepsy: in addition to reducing seizures, the KD could also significantly improve core symptoms of autism. As noted previously, children with autism and refractory epilepsy suffer poor outcomes [6], and a KD could offer dual benefits in this difficult clinical population. Consistent with a growing interest in metabolic underpinnings of neurological disorders, there has been increased basic research activity involving KDs and a dramatic increase in the number of clinical centers worldwide that administer the KD and its variants [45]–[47]. In conclusion, the present research identifies ASD as another potential major therapeutic target for metabolic strategies like the KD.
